# The microbiota‐gut‐brain axis and epilepsy from a multidisciplinary perspective: Clinical evidence and technological solutions for improvement of in vitro preclinical models

**DOI:** 10.1002/btm2.10296

**Published:** 2022-02-25

**Authors:** Federica Fusco, Simone Perottoni, Carmen Giordano, Antonella Riva, Luigi Francesco Iannone, Carmen De Caro, Emilio Russo, Diego Albani, Pasquale Striano

**Affiliations:** ^1^ Dipartimento di Chimica, Materiali e Ingegneria Chimica “Giulio Natta” Politecnico di Milano Milan Italy; ^2^ Paediatric Neurology and Muscular Disease Unit IRCCS Istituto Giannina Gaslini Genoa Italy; ^3^ Department of Neurosciences, Rehabilitation, Ophthalmology, Genetics, Maternal and Child Health Università degli Studi di Genova Genoa Italy; ^4^ Science of Health Department Magna Graecia University Catanzaro Italy; ^5^ Department of Neuroscience Istituto di Ricerche Farmacologiche Mario Negri IRCCS Milan Italy

**Keywords:** 3D culture, epilepsy, gut‐brain axis, microbiota, organ‐on‐a‐chip

## Abstract

Epilepsy is a common neurological disease characterized by the enduring predisposition of the brain to generate seizures. Among the recognized causes, a role played by the gut microbiota in epilepsy has been hypothesized and supported by new investigative approaches. To dissect the microbiota‐gut‐brain (MGB) axis involvement in epilepsy, in vitro modeling approaches arouse interest among researchers in the field. This review summarizes, first of all, the evidence of a role of the MGB axis in epilepsy by providing an overview of the recent clinical and preclinical studies and showing how dietary modification, microbiome supplementations, and hence, microbiota alterations may have an impact on seizures. Subsequently, the currently available strategies to study epilepsy on animal and in vitro models are described, focusing attention on these latter and the technological challenges for integration with already existing MGB axis models. Finally, the implementation of existing epilepsy in vitro systems is discussed, offering a complete overview of the available technological tools which may improve reliability and clinical translation of the results towards the development of innovative therapeutic approaches, taking advantage of complementary technologies.

## 1 INTRODUCTION

In the last two decades, the bidirectional connection between the gut microbiota and the brain has been extensively investigated; this link is referred to as the microbiota‐gut‐brain (MGB) axis and involves underlying biological pathways including neural, endocrine, metabolic, and immune system.^1^ Gut bacteria from different areas of the gastrointestinal (GI) tract can contribute to the central nervous system (CNS) development (e.g., neurogenesis, microglia maturation, and myelination), functions (e.g., cognition, mood, and behavior), and can also influence the pathogenesis and progression of different childhood and adult brain disorders (e.g., Parkinson's and Alzheimer's diseases, schizophrenia, epilepsy, autism spectrum disorder, and multiple sclerosis).^1^, ^2^, ^3^, ^4^, ^5^, ^6^, ^7^, ^8^

Epilepsy is a common neurological disease, affecting ~50 million people worldwide, and it is characterized by the enduring predisposition of the brain to generate seizures.^9^, ^10^ Etiology may be genetic, structural, metabolic, infectious, or immune, but up to 50% of cases are still classified as idiopathic.^9^, ^11^ The hypothesis of an interplay between the gut microbiota and epilepsy dates back to the beginning of the 20th century, with the concept of “*Bacillus Epilepticus*.”^1^, ^12^ The widespread of newer approaches (e.g., next‐generation sequencing, gnotobiology, and metabolomic) is allowing researchers to become increasingly aware of the role played by the gut microbiota in epilepsy, especially for those severe cases commonly refractory to conventional therapies, regardless of etiology.^1^, ^13^, ^14^, ^15^, ^16^

The need to dissect the biological mechanisms underlying the role of the MGB axis in epilepsy and the issues related to synergic modeling approaches for this complex interplay, rises great interest in the development of frontier technological strategies, such as organ‐on‐a‐chip (OOC) in vitro models that can be integrated into complex multi‐organ systems.

This review summarizes, first of all, the evidence of a role of the MGB axis in epilepsy, both at the clinical and pre‐clinical levels. We will illustrate available epilepsy in vitro modeling strategies, their limitations as for including microbial contribution in the experimental paradigm, and how innovative technological solutions as multi‐organ‐on‐chip platforms could eventually open up the way for a revolutionary approach in epilepsy research in the close future due to their ability to recapitulate also the microbial component for the etiopathogenesis of the disease.

## 2 THE MGB AXIS AND EPILEPSY

**FIGURE 1:**
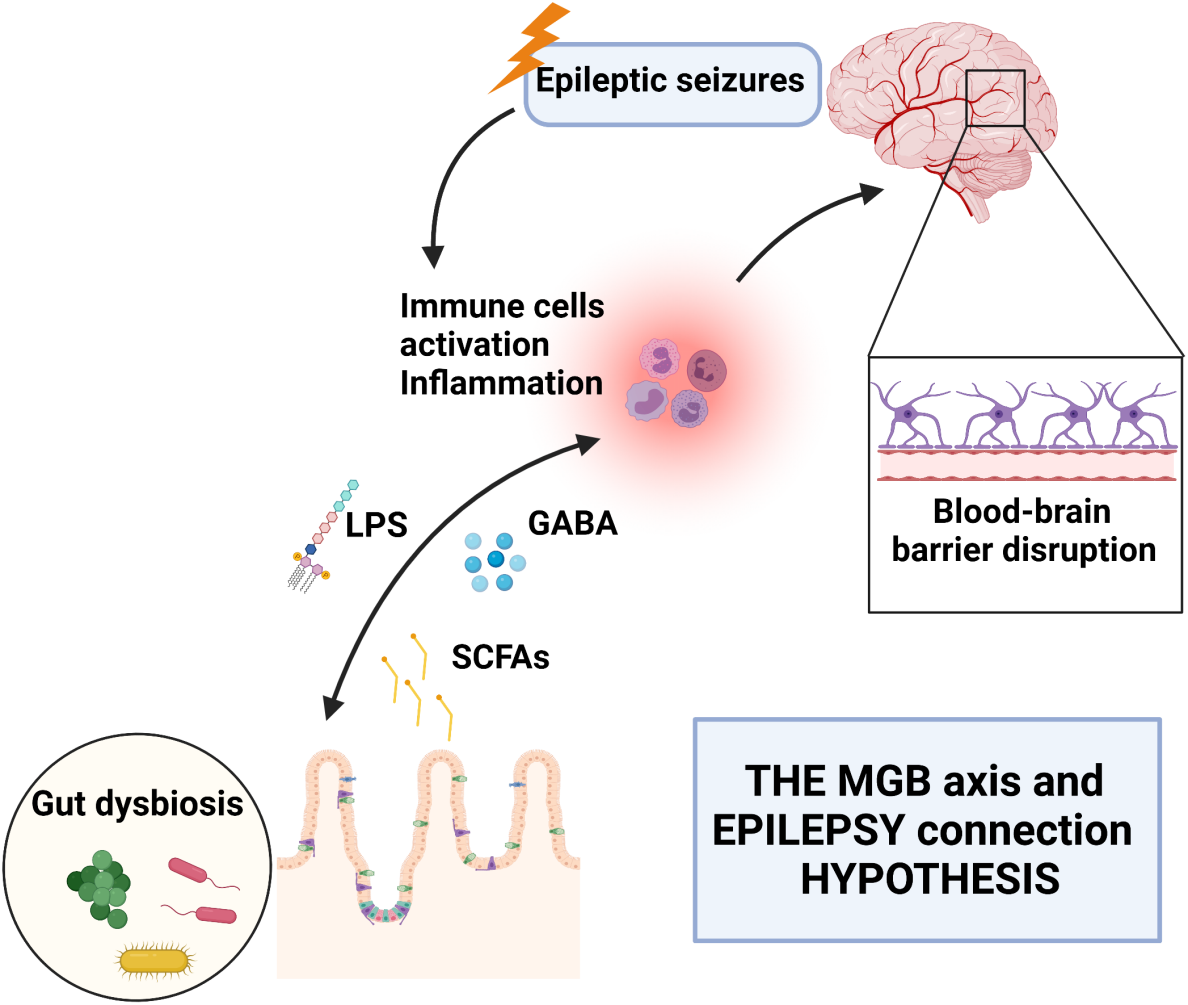
Schematic representation of the hypothesis behind the microbiota‐gut‐brain axis (MGBA) and epilepsy connection, taking into consideration the possible self‐sustaining cycle where gut dysbiosis and seizures influence each other by triggering systemic inflammation. GABA, gamma‐aminobutyric acid; LPS, lipopolysaccharides; SCFAs, short‐chain fatty acids

Starting from the antenatal and neonatal life, a wide variety of *stimuli* could act to mainly influence the composition of the host gut microbiota, which represents the thousands of microbial species inhabiting the GI tract.^17^, ^18^, ^19^, ^20^, ^21^ Increasing evidence is highlighting the systemic modulation properties of this complex GI ecosystem, potentially affecting not only immune, but also neurological processes, and leading the way to the concept of a MGB axis.^1^, ^20^ The underlying mechanism of interaction seems to dwell on a bi‐directional interplay.^22^ Alterations in the gut microbiota provide the basis for a systemic immune activation, which elicits a mirrored inflammation in the CNS, while on the other hand neurological dysfunctions may induce systemic inflammation, which in turn directly acts on the gut microbiota features.^23^, ^24^ This set of alterations can frequently be the basis for neuronal excitability dysfunction and epileptogenesis, leading to seizure susceptibility and supporting the clinical implication of the MGB axis in epilepsy.^25^, ^26^ In this scenario, a central role of the blood–brain barrier (BBB) permeability has also been extensively reported,^27^, ^28^ supporting the concept that altered BBB permeability allows gut microbiota‐produced metabolites and neuropeptides (particularly short‐chain fatty acids [SCFAs] and the inhibitory gamma‐aminobutyric acid [GABA]) to reach the CNS to exert an effect on brain functions, and so on seizures' threshold in an epileptic context (Figure 1). Other important elements of the MGB axis include the hypothalamic–pituitary–adrenal axis and the endocannabinoid system whose respective involvement in stress‐response and neuromodulation may act on CNS functioning.^29^, ^30^ Therefore, the MGB axis is a multi‐pathways network, still lacking full‐blown comprehension. Proper pre‐ and clinical studies, primarily aiming to impact these complex pathways, may lead to the development of innovative therapeutic approaches.

### 2.1 Preclinical data

Only a few preclinical studies have investigated the impact of the MGB axis on epilepsy. The attention has been mainly focused on dietary modification, microbiome supplementations, or manipulation of gut microbiota from mouse models, to evaluate pathological outcomes such as a reduction in seizures' frequency.^16^ On the other hand, the number of studies demonstrating a link between gut microbiota alterations and increased neuronal excitability and seizures is constantly increasing, shedding light on the potential mechanisms, pharmacological targets, and treatments.^31^, ^32^, ^33^, ^34^ It is worth noting that literature studies can be divided into those demonstrating a direct or an indirect impact of microbiota manipulation on epilepsy features. Among those included in the first group, some studies reported an increased susceptibility to seizures that could be transferred by fecal microbiota transplantation as well as this latter can confer seizure protection and even be used to identify molecules (microbiota metabolites) as potential treatments.^14^, ^34^, ^35^

Another example, that takes advantage of dietary treatments, is the ketogenic diet (KD), high fat and low‐carbohydrate diet, inducing ketonemia and mimicking the positive effects of fasting on CNS functioning. The KD has long been used as a non‐pharmacological treatment, mainly in the pediatric population affected by drug‐resistant epilepsies.^36^, ^37^ However, the demonstration of how the anti‐seizure effect of the KD is primarily mediated by the host gut microbiota is surprisingly recent. Olson and colleagues showed a decrease in the α‐diversity of microbial specimen, with an increase in the relative abundance of specific taxa (particularly *Akkermansia muciniphila* and *Parabacteroides merdae*) in mouse models of epilepsy treated with the KD; on the other hand, germ‐free or antibiotics‐treated mice took no advantages from the KD, supporting the hypothesis of the role of the gut microbiota in mediating the anti‐seizure effect of the KD.^14^

Regarding probiotic supplementation, only one study specifically explored the impact of probiotic supplementation in epilepsy, showing a reduction in seizures' severity in mouse models treated with a combination of *L. rhamnosus*, *L. reuteri*, and *B. infantis* as compared to control mice.^38^

Some data on how microbiota manipulation could indirectly act on epilepsy features come from studies on microbiome supplementations, namely probiotics or synbiotics (a mixture of both pro‐ and prebiotics), where evidence in epilepsy is still limited. Oral administration of a prebiotic treatment with *L. rhamnosus*, alone or in combination with *Bifidobacterium longum*, proved to modulate the CNS' expression of certain GABA mRNA receptor subunits in mouse models and this may potentially impact seizure threshold.^16^, ^39^, ^40^ Once again, Bagheri and colleagues^38^ demonstrated increased GABA brain levels in their probiotic‐treated mice, strengthening the hypothesis that gut microbiota‐produced metabolites and neurotransmitters are a key element of the MGB axis, being able to impact on seizures' threshold. Nowadays, a strong pool of research is opening to the so‐called postbiotics, which are microbial‐produced soluble factors to be orally administered.^41^, ^42^ The rationale is to directly provide the host with the final product of a symbiotic microbiota, with an application ranging from inflammatory bowel diseases to a variety of neurological diseases.^42^, ^43^ However, limitations to the “expansion” of these approaches in the clinical practice derive from the difficulties of obtaining reliable ex vivo or in vivo models, particularly when faced with the CNS which is quite inaccessible. While animal models represent the gold standard for epilepsy studies and many technological efforts have been made to improve epileptogenesis investigation directly on animals (e.g., functional magnetic resonance, electrode imaging) it is also necessary to take into consideration discrepancies in neuropathological manifestations between human and animal models. This happens, as an example, in the kindling model of acquired temporal lobe epilepsy, where symptoms and seizures are similar to those of human patients but neuropathological damages arise from different brain areas.^44^, ^45^ Moreover, electrode imaging often involves invasive techniques, offers limited information on brain activity, and has still very high variability among different animal models.^45^

### 2.2 Clinical evidence and therapeutic implications

Clinical studies comparing the gut microbiota between patients with several neuropsychiatric disorders (e.g., Alzheimer's and Parkinson's diseases, autism spectrum disorders, schizophrenia, multiple sclerosis) and healthy controls have been largely performed in the last years, suggesting differences in fecal microbiota profiles between groups.^1^ However, except etiopathogenesis, few comparative and interventional clinical studies have been published so far in human epilepsy, which is mainly focused on the impact of KD. Moreover, no randomized clinical trials on microbiota supplementation have been conducted yet.^16^ Therefore, it's worth noting that most of the microbiota‐epilepsy relationship clinical evidence demonstrated to be correlational, with only a few data on causality from available clinical studies.^46^, ^47^, ^48^ This makes preclinical studies even more important, as they could investigate specific mechanisms that may prove a causal relationship between dysbiosis and epilepsy, giving new insights to clinical research.

Among clinical evidence, gut microbiota alterations have been explored, comparing drug‐sensitive and drug‐resistant patients, investigating possible relations with the mechanisms of drug‐resistant epilepsy, as well as the influence of drugs (i.e., antibiotics) or pre‐ and probiotics on seizures occurrence via the gut microbiota. Although not consistent among studies, several CNS outcomes (e.g., seizures frequency, epilepsy‐related symptoms, quality of life [QoL] questionnaire scores) have been correlated with microbiota profiles.^30^, ^49^ Yet, it is important to highlight that available clinical studies regarding microbiota profile and human epilepsy relationship share some limitations that make it difficult to compare the obtained results. This is mainly due to differences in patient recruitment design, genomic techniques used for microbiota profiling, and sample sizes, and is the reason why there is still too little knowledge about the implications of microbiome profiles on epileptic seizures properties.^13^

A single case has been reported about fecal microbiota transplant (FMT) in a 22‐year‐old girl with a long‐lasting epilepsy status characterized by 120 seizures *per* year and Crohn's disease. During the 20 months follow‐up, a seizure‐free status was achieved and maintained without treatment with anti‐seizure medications (ASMs) discontinued post‐FMT.^50^ However, the results have not yet been replicated and a registered clinical trial on FMT in patients with epilepsy (NCT02889627) has no published results and is currently recruiting patients.^51^

The first data reporting a difference in gut microbiota profiles among drug‐sensitive, drug‐resistant patients with epilepsy and healthy controls are very recent. In 2018, Peng et al. have demonstrated a significantly increased α‐diversity, an elevated relative abundance of *Firmicutes* and *Proteobacteria* and rare‐enriched microbes (i.e., *Verrucomicrobia*) in 42 drug‐resistant patients as compared with other groups, matched to age, sex, and treatments.^15^ Notably, drug‐sensitive patients have a microbiota composition that did not statistically overlap different from healthy controls, assuming a possible bidirectional correlation between drug‐resistant mechanisms and gut microbiota that require further investigations.

A study by Xie and co‐authors has assessed the differences in microbiota profiles of 14 infants with drug‐resistant epilepsy compared with 30 age‐matched healthy controls, as well as the impact of KD on the microbiome.^52^ More in detail, the gut microbiota from drug‐resistant patients clustered distinctly from controls, indicating a difference in β‐diversity, moreover, a reduction in α‐diversity has been highlighted, although without statistical significance. Furthermore, results from this study showed KD treatment shaping the microbiota with a relative increase in *Bacteroides*, a decrease in *Proteobacteria* and without variations in *Firmicutes* and *Actinobacteria*, and overall composition comparable to the healthy controls.

However, the effects of the KD on gut microbiota were investigated in other studies in human epilepsy with different findings. Tagliabue et al. have analyzed the gut microbiota composition in six patients with glucose transporter protein 1 deficiency syndrome before and after a 6‐months KD treatment. Although the results confirmed a reduction in α‐diversity (in agreement with Xie et al.) after KD, *Firmicutes* and *Bacteroides* relative abundance were not significantly modified. However, fecal microbial profiles revealed a statistically significant increase of a bacterial group (*Desulfovibrio* spp.) supposed to be involved in the exacerbation of the inflammatory condition of the gut mucosa associated with the consumption of fats of animal origin.^53^ Notably, the same authors recently demonstrated that 1 month KD decreases SCFAs levels in responder patients.^34^ This finding is in contrast with the demonstrated efficacy and health‐promoting effects of such molecules (e.g., butyrate antiseizure effects) opening up an interesting scenario for future research.^25^, ^54^, ^55^

Zhang et al. reported reduced α‐diversity and a statistically significant increase in *Bacteroides*, associated with a decrease in *Firmicutes* and *Actinobacteria* in 20 children with drug‐resistant epilepsy after 6 months of KD.^56^ However, another study involving 20 drug‐resistant patients after 3 months of KD and 11 healthy controls showed depletion in *Actinobacteria* and *Proteobacteria* and lower α‐diversity in patients also before the dietary treatment.^57^

Only one pilot study has been performed evaluating the use of probiotic supplementations in 45 patients with drug‐resistant epilepsy. The probiotic mixture (eight different bacterial subspecies) seemed to reduce seizure frequency in the intention‐to‐treat analysis (≥50% in 28.9% patients) and increase the mean QoL score (19.2 ± 6 vs. 26.4 ± 9; *p* = 0.013) as measured in QoLIE‐10 questionnaire.^58^ However, no gut microbiota analysis has been performed preventing microbiome comparative analysis before and after treatment. Therefore, few and heterogeneous clinical studies investigating the gut microbiota have been completed so far in epilepsy, enrolling patients with different etiopathogenesis, demographic and clinical characteristics, as well as treatments strategies, making comparative analysis challenging and currently unsatisfactory.

Up to now, there are no evidence‐based treatments to target the microbiome in neurological disorders including epilepsy, although pre‐ and probiotics are already widely used in clinical practice.^58^, ^59^, ^60^, ^61^ However, some pre‐clinical evidence support the concept that gut microbiome could be necessary for KD anti‐seizure efficacy to be exploited,^14^ while several non‐antibiotic drugs including ASMs (i.e., valproate, lamotrigine, zonisamide) have been reported to shape the gut microbiota in humans.^62^ Further studies, possibly standardized and performed with the rigorous rules of pharmacological clinical trials, are mandatory to decipher the role of the MGB axis in epilepsy and discriminate if the different microbiome profiles stand causative for seizures.

## 3 OVERVIEW OF IN VIVO AND IN VITRO EPILEPSY MODELS TAKING INTO CONSIDERATION THE GUT MICROBIOTA

### 3.1 In vivo epilepsy modeling and possible technological improvements

Animal models are broadly used in epilepsy research, commonly mammals (e.g., dogs, mice, and rats), invertebrate model species, as well as both larval and adult zebrafish.

Currently, available animal models are necessary and irreplaceable to further understand the mechanisms underlying epileptogenesis in the different forms of epilepsy^63^ and to develop therapies to prevent the epileptogenic process, treat comorbidities, and drug‐resistance.

In an extensive review focused on the Zebrafish use to study the MGB axis, three key advantages have been highlighted: (1) the possibility to perform genetic manipulation with zebrafish model being an ideal system to apply recent genome‐editing technology; (2) the suitability for live in vivo imaging of host–bacteria interactions to monitor the spatial and temporal activities of immune‐signaling components by exploiting the optical transparency of the transgenic zebrafish embryo throughout its development; (3) the existence of well‐established protocols for germ‐free experiments.^64^ Moreover, Zebrafish and human microbiomes are known to have similar abundances of functional pathways despite substantial disparities in taxonomic composition.^65^ As regarding brain activity studies in vitro, innovative techniques are rapidly rising to substitute and integrate animal models, fostering innovative modeling of epilepsy and improving animal welfare.^66^

Indeed, over the last decades, high‐density microelectrode array (MEA) and microfluidic devices for small organisms, such as zebrafishes, have been extensively implemented to study seizures propagation and generation at a submillimeter scale as well as to optimize manipulation techniques. For example, microfluidics enables precise control and favorable orientation of the target through an efficient entrapment of zebrafish,^67^ whereas MEAs provide a high spatial resolution and can be used for baseline and stimulus‐evoked EEG.^68^ In vivo, MEA and microfluidic devices have been extensively reviewed in 69, 70 and 67.

Few mice studies have been performed so far using MEA with various channels, obtaining EEG from freely moving mice scalp or skull. A high‐density EEG has been proven useful in individualizing seizures source^71^ in an absence seizure model and to evaluate spatiotemporal neuronal oscillation during rapid eye movement sleep.^72^ Furthermore, 26‐channel epidural and 16‐channel scalp EEGs have been used to record and perform a spatial evaluation of visual evoked potentials.^73^, ^74^ Jonak et al. improved these methods by developing a reliable method for reusing MEA probes, allowing multisite EEG recordings without loss of quality.^68^

The first single electrode and non‐invasive long‐term EEG recording have been established only in 2013.^75^ Recently, multichannel and non‐invasive EEG recordings have been developed using embryonic zebrafish.

Indeed, Cho and colleagues have tested, in a pentylenetetrazole‐induced model of zebrafish, a non‐invasive long‐term multichannel EEG recording without embedding zebrafish in agarose. This method allowed measurements from each hemisphere of telencephalon and midbrain non‐invasively, overcoming technical limitations in telencephalon analysis.^76^ Moreover, a system named “Zebrafish Analysis Platform” has been recently designed and tested for long‐term non‐invasive high throughput multichannel electrophysiological monitoring, examining freely swimming zebrafish larvae autonomously and simultaneously within the microfluidic chamber array.^77^ Therefore, integrating the emerging technologies with different animal models of epilepsy could greatly facilitate electrophysiological monitoring, high‐throughput drug screening, and the accessibility to brain research in vivo with also a potential benefit for animal welfare. Of notice, most of these in vivo models are inclusive of an informative gut microbiota too, thus offering from this point‐of‐view a suitable tool to study also in advanced technological systems the MGB axis' implications in epilepsy.

Nevertheless, investigating a complex relationship as the MGB axis role in epilepsy is still particularly challenging in in vivo systems, where several body districts can contribute to pathological outcomes. Indeed, in vitro models with the help of innovative technologies could facilitate the clarification of molecular and mechanistic aspects at the basis of such an intricate interplay.

### 3.2 In vitro MGB axis and epilepsy modeling: Challenges for a technological integration

Several works studying epileptic activity modulation by external agents (e.g., molecules, toxins, antiepileptic drugs) couple in vivo and in vitro methods.^78^, ^79^, ^80^, ^81^, ^82^ In particular, exploiting these latter for a deeper understanding of what is observed in vivo. The preparation of brain tissues, in vitro, and their manipulation for multiple biochemical and electrochemical analysis is more accessible to study in detail the neural networks and provide the possibility to perform mechanistic studies on the brain's molecular and cellular mechanisms which is not allowed by classic in vivo models due to their intrinsic complexity.^83^ Especially for new antiseizure medications, in vitro models are considered important to link the pharmacokinetics (PK) properties, well predictable using in vivo methods, with the detection of the compounds anticonvulsant properties.^84^ Moreover, to perform neural recordings to get detailed information on brain function and synaptic plasticity, recent in vitro systems offer higher spatial resolution and higher signal‐to‐noise ratio if compared to in vivo recordings.^85^, ^86^ Organotypic hippocampal slice cultures and induced pluripotent stem cell (iPSC)‐based brain organoids are just a few examples of in vitro models that are spreading in epilepsy‐related studies.^87^

Research on the relationship between the brain‐gut axis and epilepsy is still at the preliminary stage. Recent studies on murine models have shown a close relation between gut microbiota and the occurrence of multiple types of epilepsy.^88^ However, most of the underlying mechanisms are still unknown. Progress is being made in developing strategies to investigate the bidirectional communication between the gut and the brain in general with animal models, like germ‐free mice, being essential for deepening our understanding of how microbiota alterations shape brain pathophysiology. However, these models often fail to recapitulate human scenarios due to differences in microbiota profile, molecular mechanism, immune system, and brain function like extensively reviewed by Moysidou and Owens.^89^ Therefore, the need for human reliable models led to the employment of conventional in vitro tools and the advent of more complex technological strategies for their improvement.

Recreating physiologically relevant cell culture environments and incorporating the main characteristic features of the pathophysiological in vivo conditions will help to elucidate mechanistic details of the MGB axis. To this respect, in vitro modeling with dynamic cell culture conditions has been considered the most promising approach to recapitulate the MGB complexity, also for integration with currently available animal models which are still essential in neuro‐diseases studies, especially concerning epilepsy.^90^, ^91^

Several in vitro models have been described for host‐microbiota interface recapitulation; they mimic barriers dysregulation due to inflammatory process within single organ models while is still missing a connection with other body compartments to model a systemic response.^92^, ^93^, ^94^, ^95^ So‐called “*Body‐on‐Chip*” platforms are now being developed; they are fluidic systems for dynamic complex cell culture in which multiple OOC compartments, hosting engineered microphysiological systems (MPSs), are fluidically connected. Such systems are particularly useful for in vitro PK studies and mass transport evaluation between communicating organs.^96^, ^97^, ^98^, ^99^, ^100^ Multiple modules made by microfluidic or mesofluidic chambers can host 2D or 3D tissue models which recreate the main features of a specific organ in terms of morphology, biochemical, and physical stimuli. Most advanced multi‐organ platforms were recently reviewed by Jalili‐Firoozinezhad and colleagues.^101^ From 2‐ up to 10‐organs systems were developed up to date, being gut, liver, heart, and kidney the organs that are generally modeled to study multiorgan inflammatory diseases or systemic absorption and metabolism of drugs.^100^, ^102^, ^103^, ^104^, ^105^

**FIGURE 2:**
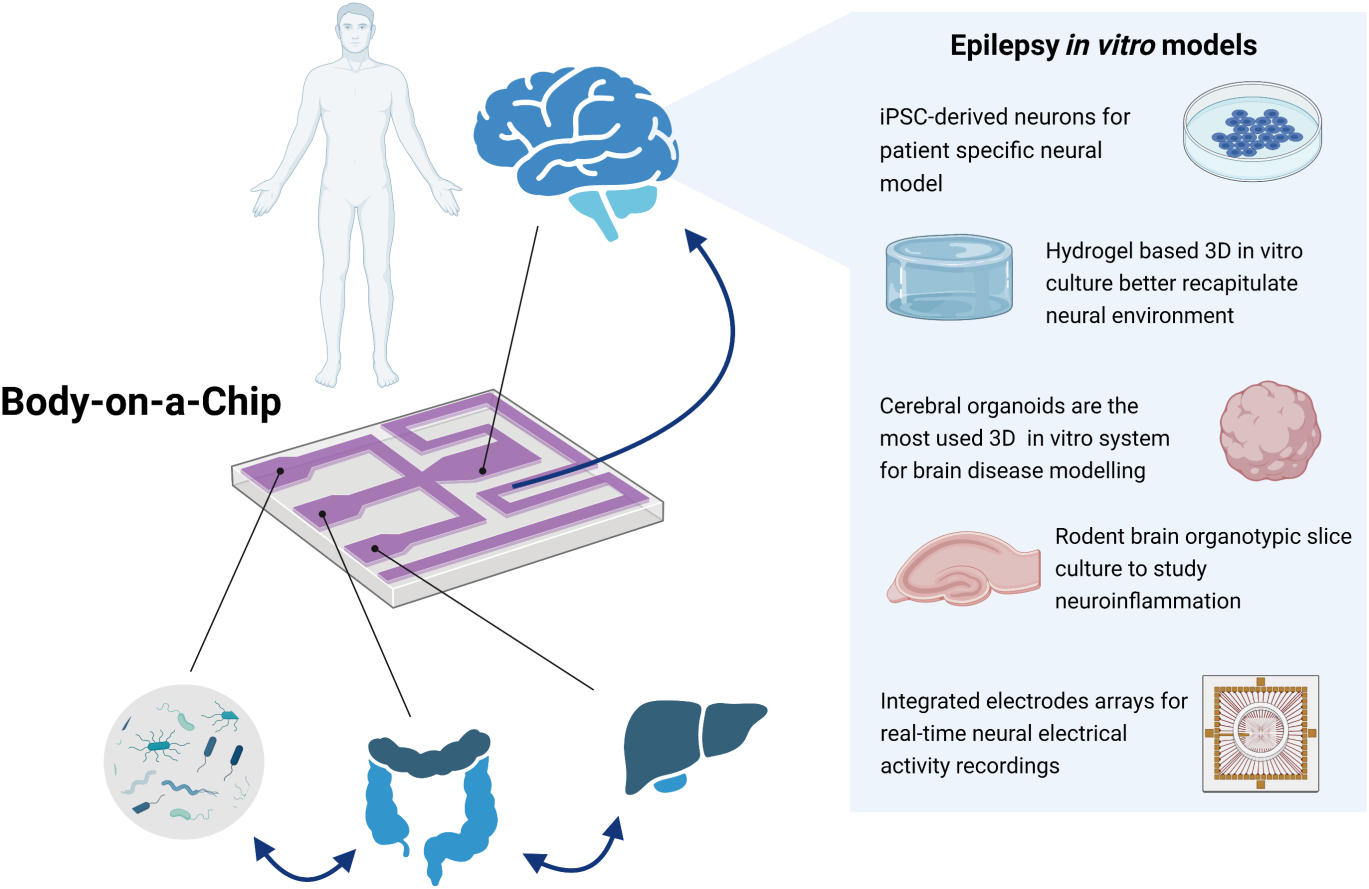
Body‐on‐a‐chip platforms are the ultimate advanced tools with the potential to give alternative systems to replace animal models in drug development. They offer pathophysiological recapitulation of the entire human body in a single device for drug pharmacokinetic (PK) and pharmacodynamics (PD) analyses in advanced interorgan systems. Microbiota, gut, and liver organ‐on‐a‐chip systems were recently developed with the demonstration of reliable inflammation scenario. While are still missing in literature examples of whole‐body neuro‐inflammatory models, body‐on‐a‐chip systems are versatile devices that can host already used in vitro neural culture models. iPSC, induced pluripotent stem cell

Recently, multi‐organ platforms for MGB axis modeling were proposed^91^, ^106^ while is still missing their integration with brain‐like compartments for epileptic seizure mimicking. This latter in particular holds many challenges and different technological strategies were explored. In the following section of this review, the technological approaches adopted by researchers for epilepsy studying and in vitro modeling are listed. A special focus will rely on the available systems and possible innovative technological strategies for an advanced brain in vitro model to be integrated within multi‐organ platforms, towards the application for epilepsy research and drug discovery also in the field of the MGB axis and epilepsy connection (Figure 2).

#### 3.2.1 Epilepsy organotypic cultures

The organotypic slice cultures of rodent brains have been used to study different aspects of neuroscience for several years.^107^ The development of genetic animal models, viral transduction techniques, time‐lapse imaging, MEA recordings, and other technological advances have allowed extensive use of organotypic culture techniques for disease and therapy‐related research.

Indeed, organotypic slice cultures are used to investigate brain lesion/regeneration and neuroinflammation as well as genetic factors, to identify the underlying mechanisms of several diseases (e.g., Parkinson's and Alzheimer's diseases), and test potential treatments.^108^, ^109^, ^110^

In the epilepsy field, organotypic cultures of rodent brain (i.e., acute hippocampal slice) have a main role to evaluate pilocarpine treatment, changes in ion concentrations, or potassium channel blockers.^111^, ^112^ These cultures approximately replicate the three‐dimensional architecture and local structure of brain cells, such as neurons, astrocytes, and microglia, as well as the neuronal connectivity and the glial‐neuronal interactions in a brain area relevant for the disease.

Magalhães and colleagues have demonstrated that mouse organotypic slice cultures gradually deprived of serum, reproduce both epileptic‐like activity and inflammatory events associated with in vivo epilepsy.^113^ Moreover, Routbort et al. have reported that the application of kainic acid to organotypic hippocampal cultures induces seizures, neuronal cell death, and mossy fiber sprouting.^114^ Furthermore, it was demonstrated the appearance of interictal‐like spikes before ictal‐like activity in in vitro preparations, resembling the epileptiform activity of in vivo epilepsy, including sensitivity to ASMs and steadily increasing seizure incidence over time, although in vitro seizure frequency and rate of epileptogenesis was higher.^115^

Organotypic cultures could be effective to study drug‐resistant seizures, identifying new anti‐seizure compounds,^116^, ^117^ as well as investigating mechanisms of epileptogenesis and neuroprotection^118^ thus reducing the need for animal models. Considering the limited viability (about 12–14 h) of acute slices obtained from human tissue, inadequate for physiological studies, human organotypic cultures could extend slices lifetime and improve efficacy and quality of studies involving human samples.^119^ Most of the advantages and limitations of using human brain slices in epilepsy research have been reviewed by Jones and colleagues in 2016.^120^

So far, only some studies have reported human organotypic cultures models useful in studying epilepsy. As an example, Fernandes and colleagues proposed a protocol for free‐floating human organotypic cultures with optimized maintenance and biological assays performance.^121^ Moreover, Wickham et al. reported increased neuronal activity and network reconfiguration in neocortical and hippocampal human brain slices.^122^ Despite the few studies using human organotypic cultures, the use of this in vitro model seems to be a useful tool in epilepsy research for the investigation of therapeutic targets and strategies and for understanding and preventing epilepsy development.

As for the relevance of organotypic slices cultures for investigating gut microbiota‐dependent molecular mechanisms, we can find in literature a few very recent examples suggesting possible interesting applications: mouse's hippocampal slices culture was performed to reveal gut microbiome‐derived metabolites having a significant impact on the mouse's circadian physiology,^123^ hippocampal slices from adult germ‐free rodents (i.e., raised in the absence of a measurable gut microbiome) were cultured to assess the electrophysiological properties of the hippocampus in a microbiome deficient animal.^124^ Despite few cases using human organotypic cultures in studying gut‐brain interplays, they can set a starting point for the investigation of therapeutic targets and strategies and understanding MGB associated epilepsy development. Noteworthy, some works in literature attribute to the organotypic term a broader set of in vitro models that we are discussing in the next paragraphs.^89^, ^125^

#### 3.2.2 Neuronal iPSC‐derived models

Human‐iPSCs represent genetically reprogrammed cells, deriving from terminally differentiated human fibroblasts or other cell types.^126^ iPSC‐derived neurons are a unique model, allowing to model features of the CNS and keeping the hallmark of the patient's genetic background.^127^ Particularly in epilepsy, where gene mutation analysis is raising into clinical practice,^128^ the possibility of implementing genome editing approaches (such as the clustered regulatory interspaced short palindromic repeats/protein 9 nuclease [CRISPR/CAS9]) to engineer iPSCs and thus generate isogenic iPSCs lines with a particular disease‐causative mutation, opens perspectives on the study of the physiopathology and possible innovative therapeutic approaches.^129^ Moreover, in an attempt to study epileptogenesis and synaptic transmission in controlled but reliable networks, recently protocols for single‐cell autoptic cultures have been developed, where a single neuron could auto‐generate synapsis to enable synaptic transmission.^130^, ^131^, ^132^

However, aiming to investigate the strict mechanism of cerebral functioning, a model including all neural subtypes is much more desirable. Hence, both excitatory and inhibitory iPSC‐derived neural cells, together with iPSC‐derived astrocytes are to be combined in a ratio resembling those in vivo.^133^ Once built, iPSC 2D cultures electrical activity could be detected with MEA systems, which transduce ionic voltage changes to easily and non‐invasively quantify electronic currents.^127^, ^133^

Another important focus lies on the possibility to differentiate iPSCs towards endothelial cells as a component of the BBB,^134^ which strictly regulate the flow of substances at the brain level. Generating an in vitro model of human BBB may be worthwhile to study brain‐penetrating molecules and the role of the BBB in certain pathways^135^ as seems unquestionable for the MGB axis. Therefore, iPSC‐derived neural and endothelial cells are innovative and, nowadays, feasible approaches to investigate CNS functioning, particularly when dealing with epilepsy which is based on electrical networks impairments. However, the complexity of neural networks cannot be reduced to 2D cultures, which anyway remain fundamental to understand differentiation pathways and implement iPSC‐derived cells into 3D and organoid models. In principle, iPSC‐derived neural and endothelial cells are valuable alternatives for studying molecular effects of gut microbiota metabolites in the field of epilepsy, also considering that they can be differentiated from a specific donor into electrophysiologically active neurons as detailed above. However, up to now, no literature data support the use of the above‐described models for this purpose.

#### 3.2.3 In vitro 3D models

2D cell cultures have some limits, triggering researchers to develop new systems capable of reproducing more reliably the cellular environment and its architecture. Also, the complexity of brain networks physiology makes it challenging to model its pathologies, including epilepsy.^45^

The value of 3D in vitro models has strongly emerged as 2D and 3D culture systems present very different environments that can influence neuronal behavior.^136^

**FIGURE 3:**
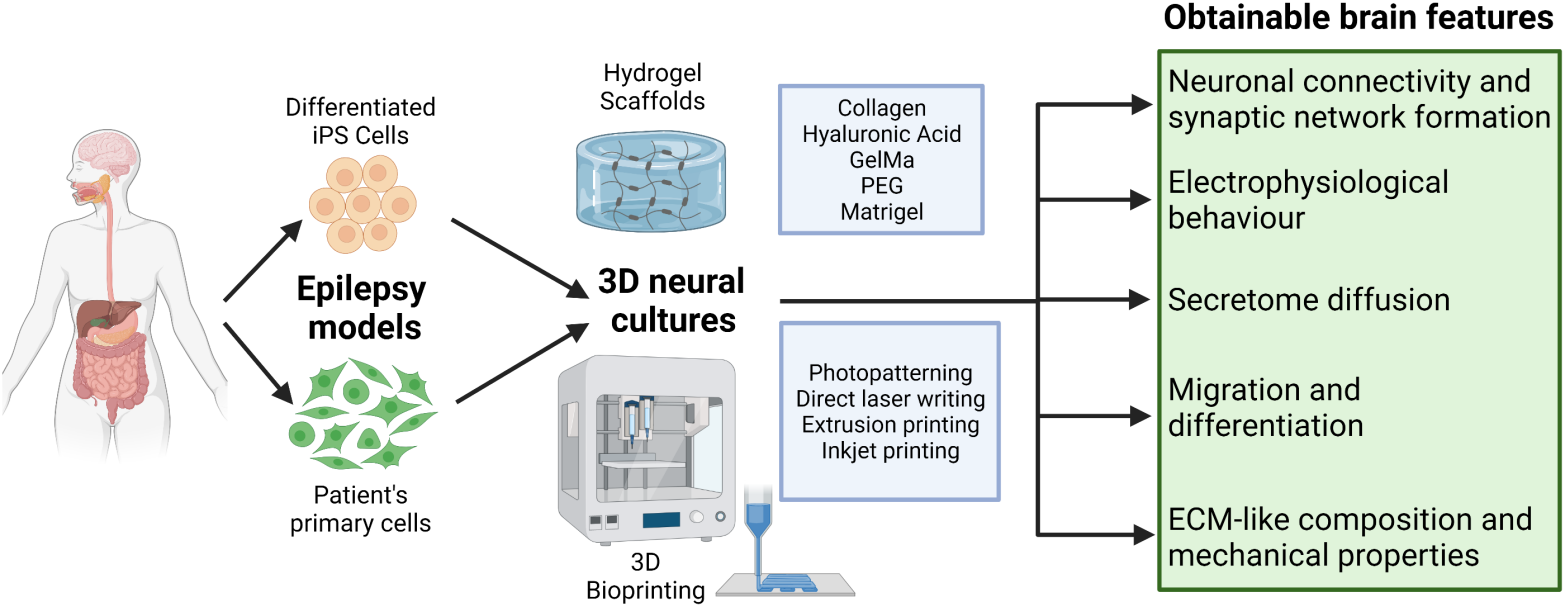
3D neural cultures suitable for epilepsy modeling, taking advantage of the use of patient's primary cell cultures of iPS‐derived differentiated neural cells. The most used technique to obtain 3D cultures is encapsulation inside cytocompatible biomaterials or scaffolds mainly composed of hydrogels that mimic the brain extracellular matrix (ECM). For this purpose, the choice of the best biomaterial is of fundamental importance (upper blue box), with compositions and pore sizes that can be adapted to the specific need. To overcome some limitations that unite hydrogels and organoids (random cell placement, small pore sizes, and necrosis due to lack of oxygen and nutrients), another innovative technique is represented by 3D bioprinting. This technology uses cytocompatible bioinks and offers different approaches to have controlled deposition of cell‐containing scaffolds (lower blue box). All 3D neural cultures, besides their limitations, could offer important brain features (green box) allowing more accurate, reliable epilepsy modeling, and analysis outputs

Thanks to the development of innovative technologies, several 3D culture systems have recently emerged, taking advantage of the use of iPSCs, biomaterials, and scaffolds to mimic the extracellular matrix (ECM), but also advanced microfabrication techniques. In this section, we will describe and put in the epilepsy context the available 3D culture technique taking advantage of cerebral organoids. Other innovative and versatile 3D neural culture techniques are bulk hydrogel systems and 3D bioprinted cultures, which are briefly summarized in Figure 3.^45^

Cerebral organoids constitute the most used 3D “top‐down” in vitro system for brain disease modeling.^137^ They are commonly derived from human PSCs (ESCs or iPSCs) with high self‐organizing property.^138^, ^139^ An organoid is obtained through embryoid body‐like aggregates floating, reproducing a differentiated 3D neural structure usually helped by natural or artificial ECM support. Cells self‐organization under specific guided or unguided protocols allows recapitulating specific CNS regions of the forebrain, midbrain, hindbrain, and retina.^138^, ^140^, ^141^

Given the neurodevelopmental nature of epilepsy‐related diseases, most of the available organoid models are centered on mimicking such disorders (e.g., tuberous sclerosis complex [TSC2], Angelman syndrome [AS], Miller‐Dieker Syndrome [MDS]), usually using patient‐derived PSCs harboring specific genetic mutations. Blair and colleagues created cortical spheroid “second‐hit” model of TSC harboring a biallelic inactivation of TSC2, which demonstrated that such 3D model displayed cells dysplasia and gliosis during neural progenitor expansion.^142^

Another disorder linked to cortical malformation is MDS (i.e., the most common disease that is linked to lissencephaly, meaning smooth and low wrinkling cerebral cortex), whose iPSCs‐derived organoid model with a heterozygous deletion involving the *LIS1* gene was developed by Iefremova and colleagues. They demonstrated that patients‐derived organoids were reduced in size, had asymmetric cell division of the ventricular zone radial glia cells, alterations of microtubules networks and cortical niche, linked to a dysregulation of N‐cadherin/β‐catenin signaling axis, thus supporting the value of the 3D organoid modeling for complex cell–cell interactions.^143^

Another work from Bershteyn et al. confirmed the utility of an organoid model from MDS patients that showed cell migration and mitotic defects in outer radial glia.^144^

Karzbrun et al. developed an organoid model to highlight the properties of the lissencephalic brain, which include reduced convolutions and elastic modulus, modified scaling, and overall mechanical instability.^145^

A human cerebral organoid from AS patients, who display increased epilepsy susceptibility, was used by Sun et al. to demonstrate that big potassium channelopathy could underlie epilepsy in AS subjects through *UBE3A* defects, leading to increased neuronal excitability and subsequent network synchronization.^146^ Finally, iPSCs‐derived human brain organoids were used by Penna et al. to study Cystatin B involvement in synaptic plasticity, as its mutation is linked to the most common form of progressive myoclonic epilepsy.^147^

Despite their huge contribution to overcoming 2D cultures limitations, organoids have some major issues that deserve attention and optimization, of which the most important is the low reproducibility, meaning significant quality, and region specificity variability.

Two recently held studies tried to overcome the reproducibility problem. Yoon and colleagues differentiated and aggregated single hPSCs generating cortical spheroids. To reach a direct differentiation, they added small molecules modulating SMAD and Wnt pathways and neuronal growth factors EGF and FGF2. They found that in 90% of differentiations, organoids were able to survive for 100 days in vitro and displayed good cytoarchitecture, spontaneous synaptic activity, cortical neuronal markers, and variability that decreased with time.^148^ Similar results, such as reproducible cell type composition upon extended culture and the presence of both radial glial stem cells and mature competent neurons, were obtained with Sivitilli et al.^149^

The absence of a vascular system in the organoid leads to the progressive necrosis of internal regions, mostly because of poor oxygen penetration, besides the fact that late development is highly dependent on blood vessels proximity, an obstacle that is stimulating a lot of interest to reach more reliable brain representation.^150^, ^151^

Another important organoid's limitation that deserves a mention is the inability to model later embryonic and fetal development, thus not making it possible to obtain full mature synapses and study circuits and connectivity, an important feature of all neurodevelopmental disorders including epilepsy‐associated ones. Together with this issue, the lack of body axes makes it difficult for the organoid to display correct migration and organization in specialized brain regions.^151^

Similar to iPSC‐derived neural models, also organoids may be simplified systems for dissecting molecular effects of gut microbiota metabolites in epilepsy research, particularly when, as in few examples detailed below,^148^, ^149^ they are capable of increasing complexity in comparison to 2D iPSC‐cultures.

#### 3.2.4 MEA technology to study epilepsy

Epilepsy in vitro studies require fine monitoring of the seizure dynamics both in physiological and pathological conditions. Generally, the quantification of epileptogenesis can be performed both by biochemical measures, biomarkers sampling or optical recording, enabling sufficient data collection for compounds screening in drug development studies.^152^ However, these methods do not give spatiotemporal details on neural network interactions; therefore, a further stage of screening is necessary by electrical recordings. Various standard electrophysiological recording techniques have been employed in epilepsy models, such as the grease gap chamber, glass‐pipetted based apparatus, carbon fiber electrodes or ion‐selective microelectrodes.^153^ While such methods allow for high‐resolution measures at a single cellular level, they are invasive and often require a complex experimental setup. The need for long‐term monitoring of electrical activity in culture tissues and simultaneous multi‐site recordings moved forward thanks to the integration of the MEA technology with organotypic cultures of rat hippocampal slices. This new approach opened to new roots for the analysis of epileptic neural interactions, as circuits, with high spatiotemporal resolution.^154^, ^155^, ^156^

MEAs devices are micrometric arrangements of a large number of metal electrodes positioned on a flat surface, used both for recording and stimulation. The basic approach consists of overlaying the brain tissue slice on top of the array ensuring close contact with the surface of the electrode. The electrodes array topography covers a relatively large area of the brain slice; hence the electrical activity across the sample can be measured at multiple points where the electrodes contact are in contact with the tissue. Commercially available MEA devices have standardized patterns with monospaced electrodes (hundreds of microns in between) converging towards the center of the chamber; this, coupled with the micrometric size of the recording tips allows for highly selective signal discrimination as well as the possibility of selecting the recording or stimulation function at single electrode level.^157^ The shape and structure of the microelectrodes have been proposed in many forms according to the specific application or, for improvement of the recording performances. One example is represented by protruding 3D electrodes, for better slice tissue penetration near the living neurons, which demonstrated that the amplitude of evoked potential responses was significantly larger than those obtained with planar MEAs.^158^ Moreover, the array topography can be easily customized as well. Epileptic seizures were found to spontaneously arise from a specific area on hippocampal slices and many models recently tried to predict the complex spatiotemporal network propagation.^159^, ^160^, ^161^ To follow such fine and complex signal distribution, some groups developed custom‐designed electrodes arrays with tissue‐conformal configurations, creating epilepsy‐specific high‐density MEAs that conform to the cytoarchitecture of the nervous tissue of interest.^162^, ^163^

Electrographic screening, using the described approaches allowed us to achieve interesting findings in the identification of antiseizure medications, giving more information about the effects of different drugs on epileptogenesis^152^ as well as about epileptic seizures dynamics both in vivo and in vitro.^164^, ^165^

In the last decade, big efforts have been made in developing MEA devices for recording the spiking activity of live neurons cultured in vitro, with the final aim of more reproducible live neuronal networks.^157^, ^166^, ^167^ Indeed the non‐invasive nature of MEA recordings already showed significant improvements, over traditional long‐term cultures of brain slices, for synaptic connectivity in cultured hippocampal networks.^168^ Moreover, this approach is particularly useful when investigating the anti‐seizure mechanisms of drugs that may not reach the inner region of the prepared slice efficiently; indeed it is possible to study their effect directly on cultured neurons by measuring seizures activity.^157^ To enhance the development of specific patterns that lead to the formation of neural networks at the culture surface, innovative works proposed the use of micro‐printing and soft lithography fabrication techniques with cell patterning approaches. Most recently, specific geometric substrates were coupled to fully planar MEAs as a new technological solution for a better translation of MEA technology in advanced culture systems and also in vivo applications.^169^, ^170^

One of the major issues in MEA based epilepsy in vitro studies is the long‐term maintenance of the brain sample survival within the recording. Maintaining the brain slice oxygenated and hydrated for long periods, and avoiding in this way hypoxia and necrosis, is challenging in traditional MEAs. In the next section, we will explore the impact of dynamic culture conditions on brain and epilepsy in vitro studies with a focus on the development of dynamic perfused chambers for long period neural activity recordings. Moreover, the dynamic solution with OOC devices may be also the key to build up a bi‐modal platform, where one chamber hosts already available dynamic models of the gut microbiota or capable to culture a selected bacterial strain,^171^, ^172^ and the second chamber hosts an in vitro model of epilepsy comprehensive of MEA recording. Up to date, literature lacks of works employing MEAs for in vitro detection of the electrical neural activity related to gut dysbiosis with only one recent example, to our knowledge, where electrophysiological properties of the hippocampus have been assessed in a microbiome deficient animal.^124^

In the last part of the review, we will deepen the description of OOC technology applied to epilepsy research, keeping in mind the possibility offered by microfluidic solutions to add the complexity of microbiota and gut to the experimental set‐up.

#### 3.2.5 Advanced long‐term dynamic culture and brain‐on‐a‐chip systems

The lack of flow perfusion represents one of the main limiting factors for durable, long‐term cultures of brain organoids or brain slices in neurotoxicity in vitro studies and this is applied also to epilepsy and MGB axis research. Moreover, to study systemic inflammation, static culture models cannot replicate the physiologic or pathological transport of molecules which are conveyed by body fluids, in vivo. Continuous and long‐term fluid perfusion allows for experimental models of continuous and prolonged interactive metabolic cross‐talk between organ systems which have been demonstrated a significant advance in modeling the human gut‐brain axis in the context of neurodegenerative diseases in vitro.^106^

Dynamic culture systems not only aim to improve the diffusion of oxygen and nutrients to the cells but also to reach high‐throughput outcomes after a long period (Table 1). At the same time, maintaining the sample vital for long periods represents a challenge as well as integrating the culturing environment with micro‐fabricated structures like MEAs.

**TABLE 1:** List of currently used systems for flow perfused long‐term cultures of brain organoids and brain slices applicable to epilepsy research

Application	System	Description	Outcome	Reference
Brain organoids generation Brain region‐specific organoids generation from hiPSCs (e.g., forebrain, midbrain, and hypothalamus) recapitulate key features of the developing brain	Orbital shakers	Standard multi‐well plates on shaker device for circular shaking motion	Cost‐effective set up and protocols Flow velocity profiles and shear stress distribution allowed for correct development of the organoids in space and time	173
	Miniaturized spinning bioreactor (SpinΩ)	3D printed 12‐well mini‐bioreactor with automated gears to provide suspension environment inside the wells and favor oxygen delivery to the organoids	Embryonic organoids generation within a low‐shear environment Enhanced molecules mixing in a reduced culture volume High throughput and reproducibility. Adaptable to complex multiorgan fluidic platforms	174
	Perfused system	Multichannel microfluidic PDMS based chip for Brain‐organoid‐on‐a‐chip model	Improved cortical development compared with static cultures Organoid's prolonged culture is possible Low‐cost and easy to operate In situ tracking and real‐time imaging	175, 176
Brain slices culture	Perfusion systems with porous substrates	Porous membrane, as support to the slice sample, separates the gas perfusion chamber from the medium perfusion chamber	Flowing medium in the perfusion chamber reaches the tissue directly placed on top of the array. A thin sheath of fluid on top of the slice guarantees the maximum oxygen rate to the cells. Integration with MEA directly fabricated on porous substrates Suitable for short culture periods only	177, 178
	Mini‐well perfused device	PDMS microfluidic perfusion system with large circular chambers for brain slice hosting	Controlled brain slice perfusion and oxygenation Stable organotypic culture on MEAs High throughput screening of slice's electrical activity	179

Abbreviations: hiPSCs, human‐induced pluripotent stem cells; MEA, microelectrode array; PDMS, polydymethilsyloxan.

In the context of more complex fluidic systems like the already mentioned multiorgan platforms, in particular for modeling the microbiota‐immune‐CNS interaction, having a highly controlled dynamic in vitro system is fundamental to model the metabolites and neurotoxins transport between different compartments.

From an engineering point of view, modulating the velocity profiles and the shear stress acting on the barrier is as crucial as challenging to achieve. Moreover, as concerning epilepsy and body fluid flows, it was observed dynamic ictal perfusion changes during temporal lobe epilepsy.^180^ Tuning the flowrates within the neurons culture microenvironment with physiologically relevant velocity levels would add a further level of complexity to epilepsy in vitro models. In such a scenario, brain‐on‐chip (BoC) devices represent an innovative approach for integrating biology and bioengineering. They consist of microfabricated platforms that reproduce the CNS physiological microenvironment and tissue mechanical properties and responses to stimuli, allowing the development of several devices for specific neurological disease modeling, but also the study of brain networks in their complexity.

The ideal BoC device could potentially include several innovative technologies, gaining all the advantages of dynamic perfusion, 3D culturing, and electrophysiological recordings.

Moreover, the “on‐chip” approach offers flexibility, robustness, and the high‐throughput monitoring and stimulation of neural cells.^181^, ^182^

Importantly, specific requirements in terms of cell types and biological outcomes to be assessed in the BoC, influence enormously both the design and fabrication process of the device.

There are some fundamental elements for BoCs design and manufacturing: microchannels, that can be used as neurite growth guide, as scaffolds, or basically to provide perfusion and biochemical gradients; microchambers, to allow spatial separation between different cell types or heterogeneous tissues formation; ECM components for three‐dimensionality representation; electroactive components such as MEAs for stimulation and recording. All those elements are connected to specific functional features as dynamic mechanical stress, mass transport of solutes, analysis of neural electrical activity, and distribution of biochemical cues.^183^, ^184^, ^185^, ^186^, ^187^, ^188^

As for biological models to be included in the BoC, the device can be adapted to a vast number of configurations, ranging from single‐cell cultures to neuronal circuits, co‐culture of neurons and glial cells, or even the integration between glial/neuronal cells and BBB components. In this sense, 3D techniques such as 3D‐printed hydrogels offer the advantage of assembling heterogeneous biological systems mimicking their connections and communication in a more realistic environment.^183^, ^186^, ^189^ Moreover, it is worth taking into consideration the integration with BBB‐on‐a‐chip systems for inflammation‐epilepsy studies. BBB damage following peripheral inflammation seems to be related to epileptogenesis initiation or worsening. It is not clear whether BBB impairment is a cause or a consequence of epileptic seizures. However recent findings suggested that molecules extravasation, like albumin, into brain parenchyma due to BBB dysfunction can induce epileptogenesis. From here, the importance of studying the mass transport phenomena in dynamic culture models with the help of advanced tools. Hopefully, understanding complex phenomena related to BBB alterations, in systemic inflammation models, like the MGB axis model, in response to seizures and epilepsy can open to novel treatment strategies.^190^, ^191^

Different BoC devices have been developed in the last decade, having one or more of the aforementioned features and various disease study applications (e.g., Alzheimer's disease, Parkinson's disease, traumatic brain injury).^175^, ^192^, ^193^, ^194^ However, specific development of “epilepsy‐on‐chip” systems is still poorly treated, probably due to the challenge of modeling a pathology that must necessarily consider both the establishment of neuronal networks and the possibility of stimulating and recording with extreme efficiency.

Section 4 will be dedicated to an overview on the potential development of epilepsy‐specific BoCs, with a focus on some interesting examples of brain devices adaptable to epilepsy, and description of the first complete epilepsy‐on‐chips optimized for drug screening.

## 4 TOWARDS CLINICAL TRANSLATION: TECHNOLOGICAL INTEGRATION WITH IN VITRO EPILEPSY MODELS (“EPILEPSY‐ON‐CHIP”)

OOC technology is an emerging field in bioengineering aiming to fill the gaps in drug screening by recreating human tissue models for more reliable predictions of drugs efficacy and safety in support of clinical trials.^96^, ^195^ The main characteristic feature of a specific organ is recreated within a sophisticated complex microenvironment using advanced technological approaches, some of which we recapitulated in this review. Up to date, several organs scenarios have been modeled in innovative OOCs: the microbiota‐gut interface, the BBB, or the lung microenvironment are just a few examples.^196^, ^197^ In this review, we already went through OOCs applied to Brain physiology or disease modeling which are called BoCs and their main technological features.

As concerning epilepsy, in vitro modeling is recently approaching advanced technological routes (Table 2). However, there are still missing examples of MPSs recapitulating the dynamic interconnection between epileptic brain regions and other body compartments in vitro.

**TABLE 2:** Overview of the combined technological approaches implemented during the last two decades into in vitro models of epilepsy and other neurological diseases adaptable to epilepsy studies

Reference	Organotypic culture	Cell cultures		2D	3D		Electrical recordings		Dynamic culture	BoC
		Primary neural cultures	Neural iPSC derived cultures		Organoids	Hydrogel based 3D neural cultures	Single electrode	MEA		
154	+							+	+	
155	+							+		
156	+							+	+	
114	+						+			
158	+							+		
168		+		+				+		
162	+							+		
198		+		+				+		
199	+							+	+	+
115	+						+		+	
169	+							+	+	
166		+		+				+		
200		+				+	+			
201		+				+		+		
194	+								+	+
202		+				+	+			
187		+				+			+	+
175			+		+				+	+
203	+							+	+	+
193		+		+					+	
204			+			+				
189		+		+				+		
113	+						+			
186		+				+			+	+
174			+		+				+	
176			+		+				+	+
185		+		+					+	
157	+	+		+				+		
179	+							+	+	+
147			+		+					
184		+		+				+	+	
167		+		+				+		
205			+	+				+	+	+

Abbreviations: BoC, brain‐on‐chip; iPSC, human‐induced pluripotent stem cell; MEA, microelectrode array.

The first example of a complex dynamic system for continuous electrophysiological studies was presented, in 1997, by Stoppini and co‐authors.^154^ They developed a multi recording device (Physiocard®) where the MEA was integrated on top of a dedicated perfusion chamber. The recording system allowed for electrical stimulation and electrophysiological recordings from organotypic cultures of rat hippocampus for several hours. At that time, the authors proposed their simple and user‐friendly device as a promising tool for various CNS applications, among which epilepsy studies. The main innovative aspect was the possibility of performing controlled drug delivery by a perfusion system and testing the effects of the specific molecule on synaptic activity. This system represented a starting point for combining different technological aspects and physical stimulations in a controlled culture environment towards the concept of OOC devices. The advent of soft lithography and polydymethilsyloxan (PDMS) for fabrication of microfluidic chips for single‐cell studies led to the development of microsystems for organotypic chronic electrical monitoring. The first example of microfluidics applied to organotypic MEA assays was reported by Berdichevsky et al. Their device allowed for hippocampal slices culture up to several weeks inside PDMS mini‐wells while recording synaptic activity by a traditional planar MEA, which was successfully integrated inside the microsystem. Interestingly, this represents the first case of microchannels incorporated into the well for brain slice maintenance. The micrometric dimension of the channels and the controlled flow velocity led to axonal growth and alignment along the channel direction.^199^ This latter phenomenon was also reported by Shen and co‐authors who proposed a neuro fluidic micro‐device showing the influence of microfluidic constrain on the functional neural connectivity, which was measured by an integrated MEA inside the microchamber.^184^

Liu and colleagues recently proposed μflow‐MEA, the first epilepsy‐on‐a‐chip system for ASMs discovery.^179^ μflow‐MEA is the most evolved example of the integration of organotypic culture, MEA, and microfluidics. It was born from a previously developed perfused drop microfluidic device.^203^ It consists of a network of microfluidic channels for controlled nutrient uptakes to the brain slice laying inside circular micro‐wells. The PDMS‐based microfluidic system was bonded to a simplified metal patterned MEA without the need for an insulation process, which makes the device cheaper and easier to fabricate without compromising the sensitive detection of seizure‐like activity. The authors demonstrated that μflow‐MEA based chronic electrophysiological recordings, used for screening of hippocampal RTKs inhibitors, reflected the results obtained from the analysis of typical epilepsy biomarkers. In detail, μflow‐MEA demonstrated to be suitable for epileptogenesis detection both at electrical (i.e., number and duration of seizure‐like events) and biochemical level (i.e., lactate and LDH level measurements). Moreover, this microfluidic platform allowed the efficient screening of 12 potential drugs, confirming its validity for high‐throughput ASMs discovery.

This epilepsy‐on‐a‐chip drug screening system opens new routes towards a deeper comprehension of the complex signaling pathways involved in epileptogenesis, being a promising starting point for always more reliable drug screening steps in support of clinical trials. However, it is one of the few available BoCs that shown to sustain drug screening for epilepsy treatment and still exploits the use of animal experimentation for organotypic slices obtainment.

Valid alternatives can be found in recently proposed BoCs for neurological disease applications which offer technological strategies (e.g., microchambers, compartmentalization, advanced MEA) that can be exploited for integration of epilepsy models.

Park et al. developed an in vitro model of Alzheimer's disease.^175^ The microfluidic chip contained micro‐wells in which the formation of homogeneous neurospheroids occurred. An osmotic micropump system connected to the outlet provided a continuous flow of medium that contained oxygen and nutrients. This microfluidic device retains two in vivo brain characteristics, the 3D cytoarchitecture and the physiological interstitial flow. Moreover, Wang and colleagues developed a simple and robust micro‐device that allows generating hiPSCs‐derived brain organoids in a controlled manner.^176^ Through their platform, they examined the features of neural differentiation, brain regionalization, and the cortical organization in the brain organoids.

Soscia et al.^189^ proposed a platform to reproduce complex neuronal cultures and record brain cells excitability. Their system has the peculiarity of having a removable insert to separate distinct cell populations. The MEA was divided into two different regions to study two distinct populations of neurons: cortical and hippocampal. The possibility of having separate regions for specific cell culture and MEA recording is the point of strength of the most recent developed BoC device for epileptic seizures modeling by Pelkonen et al.^205^ The MEMO platform allows for culturing human pluripotent stem cells in three distinct areas and can model the network‐to‐network axonal connections through microtunnels. The spontaneous neuronal network activities were then monitored with the integrated MEAs. This configuration mimics both local and circuitry functionality of the brain enabling to study of the effect of abnormal seizure activity.

The MEA integration inside these microsystems is perhaps one of the most challenging aspects. One interesting solution was proposed by Sharf et al.^206^ They reported a system for monitoring the electrical activity generated by multi‐cellular networks in a non‐contact configuration. In this way, cells can be grown on conventional cell culture substrates and the recording electrodes array can probe different cultures in succession, without degrading its sensitive electronic surface. Moreover, this configuration is particularly suitable for micro‐channels thanks to the micrometric distance between the cells and the MEA. Hence, considering the available epilepsy models and the technologies described above, it is evident that the integration of the biological aspects of brain cells culturing with the technologies that bioengineering can provide, including an OOC solution to model the microbiota‐gut compartment, constitutes the new frontier for reliable epilepsy disease modeling, contributing to the clinical translation also in a multi‐organ approach.

## 5 CONCLUSION: PERSPECTIVES AND CHALLENGES FOR INNOVATIVE EPILEPSY RESEARCH WHEN TAKING INTO ACCOUNT THE MGB AXIS PARADIGM

The microbiome plays a significant role in the health status of its host. Since the gut microbiota was first proposed to influence human health over one century ago, our understanding of its role has immensely improved. During the past decade, the scientific community has made outstanding technological advances in microbiome characterization. Through basic science, translational, and clinical research, we have now gained insight into gut‐brain communication and enhanced our understanding of the complex multi‐directional relationships that exist between gut, host, and environment. In particular, multi‐omics approaches based on the analysis of different body fluids and tissues with various profiling platforms have the potential to provide deeper insights into MGB axis disorders, including response to treatment and the contribution of environmental factors. Therefore, biomarker discovery experiments based on profiling approaches facilitated by recent technical development are likely to make a great contribution to uncovering disease mechanisms in complex neuropsychiatric disorders. Serious consideration should be also given to the concurrent analysis of global metabolic changes peripherally (e.g., in the blood) and centrally (in cerebrospinal fluid, CSF) to establish how closely abnormalities measurable in the blood are correlated to changes in the brain. These researches could eventually lead to targeted therapy for the microbiota, preventing alterations or acting to restore normal intestinal flora.

However, future research on gut microbiota in human epilepsy and animal models are needed to develop any microbiome‐specific therapeutic strategy and establish whether microbe‐based treatments can be effectively and securely used for clinical improvement of seizure incidence, severity, and related disorders. In particular, studies are needed to develop validated methodology to study the functional role of other microorganisms also present in the gut microbiota, such as fungi, protists, phages, and archaea, and their interplay among them and with the host. Also, ongoing microbiome/microbiota mapping projects must clarify or confirm the global inter‐individual differences and detect microbiological profiles to be used as healthy control. Finally, future studies will be able to improve our knowledge and open up new therapeutic options through manipulation of the gut microbiota by dietary changes, specific pre‐ and probiotic supplements, or FMT. Elucidating the connection between the MGB axis and epilepsy could lead to the discovery of useful biomarkers and advance knowledge on the complex mechanisms underlying epileptogenesis and epilepsy themselves.

From the technological point of view, even though in vitro models of the main players in the MGB axis were recently successfully developed they still lack a robust multi‐organ crosstalk. Recent body‐on‐chip systems represent valuable tools for bench studies on such interconnected body compartments. BOC technology offers a novel approach for mimicking the brain micro‐environmental conditions to study neural response to drugs, by including complex co‐culture 3D models with tunable interfaces between different anatomical compartments. To further integrate the epilepsy scenario within the “brain” compartment of an MGB axis platform, advanced technological approaches are nowadays available with many possible configurations that were explored in the last decades. Hippocampal organotypic culture is the gold standard for the investigation of neural networks involved in epileptic seizures; the technological advancement led to consistent improvement of the experimental platforms, which were complex and allowed for short‐term analysis only with scarce reproducibility. The integration and combination of short‐scale fluidic systems, smart materials, and specific microelectrodes arrays, by accessible and cost‐effective fabrication techniques, permits the construction of tailored cell culture devices with controllable micro‐environmental conditions resembling the epileptic pathophysiological scenario. Integrating a pathophysiological epilepsy model within MGB axis in vitro platforms would represent a challenge both from the technological and biological points of view. Examples are (i) culturing of different heterogeneous cellular models inside interconnected culture chambers by systemically conveying culture media with common composition; (ii) performing organotypic culture or 3D culture inside a fluidic platform with other different organ models that may be 2D or 3D barrier models to recapitulate the systemic inflammatory process; (iii) introducing access points to the platform for sample manipulation; or (iv) the implementation of complex electrodes arrays inside epilepsy/brain compartment by fast production techniques.

In conclusion, besides many challenges to face, the promising outcomes from the newborn epilepsy‐on‐chip technology are supportive of the feasibility of new strategies for reliable epilepsy in vitro models to be integrated inside complex dynamic multi organs platforms, with a mid‐term impact also on the molecular mechanism, drug and biomarker discovery and ultimately clinical translation.

## CONFLICT OF INTEREST

E.R. has received speaker fees or funding from, and has participated in advisory boards for, Angelini, Arvelle Therapeutics, Eisai, Kolfarma, Pfizer, GW Pharmaceuticals, UCB, Lundbeck, Italian Ministry of Health (MoH), Italian Ministry of University and Research and the Italian Medicine Agency (AIFA). A.R. has received honoraria from Kolfarma s.r.l and Proveca Pharma Ltd. P.S. has received speaker fees and participated at advisory boards for Biomarin, Zogenyx, GW Pharmaceuticals, and has received research funding by ENECTA SV, GW Pharmaceuticals, Kolfarma srl., Eisai.

## AUTHOR CONTRIBUTIONS

**Federica Fusco:** Conceptualization (lead); writing – original draft (lead); writing – review and editing (lead). **Simone Perottoni:** Conceptualization (lead); writing – original draft (lead); writing – review and editing (lead). **Carmen Giordano:** Conceptualization (equal); writing – original draft (supporting); writing – review and editing (equal). **Antonella Riva:** Writing – original draft (equal); writing – review and editing (supporting). **Luigi Francesco Iannone:** Writing – original draft (equal); writing – review and editing (supporting). **Carmen De Caro:** Writing – original draft (supporting); writing – review and editing (supporting). **Emilio Russo:** Conceptualization (equal); writing – original draft (supporting); writing – review and editing (supporting). **Diego Albani:** Conceptualization (equal); supervision (equal); writing – review and editing (equal). **Pasquale Striano:** Conceptualization (equal); project administration (lead); supervision (lead); writing – original draft (supporting); writing – review and editing (equal).

### PEER REVIEW

The peer review history for this article is available at https://publons.com/publon/10.1002/btm2.10296.

## Data Availability

Data sharing is not applicable to this article as no datasets were generated or analyzed during the current study.
